# Efficacy and safety of CTLA-4, PD-1 and LAG-3 immune checkpoint inhibitors as monotherapy and combination therapy in advanced melanoma: A systematic review and meta-analysis

**DOI:** 10.17179/excli2025-9096

**Published:** 2026-01-30

**Authors:** Osama Omar Khan

**Affiliations:** 1 Faculty of Medicine and Health Sciences, University of Buckingham, Buckingham, United Kingdom

**Keywords:** advanced melanoma, immune checkpoint inhibitors, PD-1, CTLA-4, LAG-3, combination immunotherapy

## Abstract

Advanced unresectable melanoma carries a poor prognosis, with minimal benefit from chemotherapy and limited responsiveness to radiotherapy. The emergence of immune checkpoint inhibitors (ICIs) targeting PD-1, CTLA-4, and LAG-3 has transformed treatment outcomes; however, their comparative efficacy and safety remain unclear. This systematic review and meta-analysis evaluated and compared the efficacy and safety of PD-1 and CTLA-4 monotherapies against traditional systemic therapies, as well as the dual regimens PD-1 + CTLA-4 and PD-1 + LAG-3 against their respective monotherapies, in patients with advanced unresectable melanoma. A comprehensive search of PubMed and the Cochrane CENTRAL database was conducted on March 18, 2025, for randomized controlled trials comparing ICIs against conventional therapies or other ICI regimens. Primary endpoints included overall survival (OS), progression-free survival (PFS), objective response rate (ORR), and grade ≥ 3 adverse events (AEs). A random-effects meta-analysis was performed, and risk of bias was assessed using the Cochrane RoB 2.0 tool. Eleven randomized controlled trials (n = 4,111) met the inclusion criteria. The PD-1 + CTLA-4 combination demonstrated the greatest clinical benefit, significantly improving OS (HR = 0.59), PFS (HR = 0.45), and ORR (RR = 3.11) compared with monotherapy, but was associated with a higher incidence of grade ≥ 3 AEs (RR = 2.14). The PD-1 + LAG-3 regimen showed a moderate yet statistically significant advantage in efficacy over PD-1 monotherapy (OS HR = 0.80) while maintaining better tolerability. PD-1 monotherapy demonstrated greater efficacy and a more favorable safety profile than CTLA-4 monotherapy when each was compared with traditional therapy. In conclusion, PD-1 + CTLA-4 offers the most substantial therapeutic improvement but with considerable toxicity, whereas PD-1 + LAG-3 provides a more balanced efficacy-safety profile. PD-1 monotherapy remains the safest option, though less effective than combination strategies. These findings highlight the evolving role of combination immunotherapies and the potential clinical value of LAG-3 as a novel checkpoint target in melanoma management.

See also the graphical abstract(Fig. 1).

## Introduction

Melanoma arises from malignant transformation of melanocytes, which originate from neural crest. They are not only limited to the skin but can also develop in the other parts of body where neural crest cells migrate, such as mucosal membranes, brain and uveal tract of the eye. This transformation results from the accumulation of genetic mutations leading to uncontrolled proliferation of the malignant melanocytes. Contributing factors include genetic predisposition, ultraviolet radiation and other environmental influences (Heistein et al., 2025[8]). As a result, melanoma has become a global health concern, with more than 330,000 new cases and 58,000 deaths were reported worldwide in 2022. Melanoma continues to rise in high income regions like Australia, Europe and North America (Ferlay et al., 2024[3]).

Early diagnosis of melanoma at stage 0 or 1 is often curable with surgical resection, providing five-year survival rates in approximately 97 % of patients. However, survival outcomes deteriorate significantly in advanced stage of the disease (unresectable or metastatic melanoma), five-year survival rate in advanced melanoma is around 30 % (Heistein et al., 2025[8]; Tonella et al., 2021[26]).

Traditional treatments, such as chemotherapy (e.g., dacarbazine) and radiotherapy, have not provided consistent survival benefits in advanced unresectable melanoma, primarily serving palliative purposes (Kalal et al., 2017[13]). The introduction of targeted therapies, including BRAF and MEK inhibitors, led to notable improvements in patients with specific mutations. However, these benefits are often short-lived due to acquired resistance, mainly through MAPK pathway reactivation or alternative survival mechanisms, resulting in disease progression (Kakadia et al., 2018[12]).

Over the past decade, immune checkpoint inhibitors particularly those targeting PD-1, CTLA-4, and LAG-3 have significantly improved overall and progression-free survival in patients with advanced unresectable melanoma. The immune system can recognize and eliminate abnormal cells, including tumors, through T-cell activation. This process requires two signals: antigen recognition via the T-cell receptor (TCR) and co-stimulation, typically through CD28 binding to CD80/CD86 on antigen-presenting cells (APCs). To avoid excessive immune activation, this process is regulated by immune checkpoints (Vignali et al., 2008[28]). CTLA-4 (cytotoxic T-lymphocyte associated protein-4) is an inhibitory receptor on activated T-cells that binds CD80/CD86 with higher affinity than CD28, blocking co-stimulation and dampening T-cell activation. CTLA-4 inhibitors (e.g., ipilimumab) are IgG1 monoclonal antibodies that block this interaction, allowing CD28-mediated signaling and full T-cell activation (Rausch and Hastings, 2017[18]).

PD-1 (programmed cell death protein-1) is another inhibitory receptor on activated T-cells. It binds PD-L1 or PD-L2, which are often overexpressed on tumor and antigen-presenting cells. Persistent PD-1 signaling leads to T-cell exhaustion, marked by reduced proliferation, cytokine production, and cytotoxic activity, enabling tumor immune evasion. PD-1 inhibitors (e.g., nivolumab, pembrolizumab) are monoclonal antibodies that block PD-1, restoring T-cell function and enhancing tumor elimination (Rausch and Hastings, 2017[18]).

All antigen-presenting cells (APCs), including dendritic cells, macrophages, and B-cells, express MHC class II molecules, which present extracellular antigens to CD4+ T-helper cells, leading to their activation. Upon recognizing an antigen-MHC-II complex via the T-cell receptor (TCR) and receiving co-stimulatory signals, CD4+ T-cells proliferate and secrete cytokines that drive broader immune responses, including B-cell antibody production and indirect activation of cytotoxic T-cells. Through these pathways, CD4+ T-cell activation contributes to the detection and elimination of abnormal cells, including tumors (Haabeth et al., 2014[4]).

LAG-3 (lymphocyte activation gene-3) is an inhibitory receptor on activated T-cells. It binds MHC class II with higher affinity than CD4, delivering inhibitory signals that reduce T-cell proliferation, cytokine release, and effector function. In the tumor microenvironment, LAG-3 is often co-expressed with other inhibitory receptors like PD-1, and their combined activity leads to T-cell exhaustion and immune evasion (Ruffo et al., 2019[24]). LAG-3 inhibitors (e.g., relatlimab) are monoclonal antibodies that block this interaction, sustaining T-cell activation and enhancing anti-tumor responses (Huo et al., 2022[11]).

### Current evidence

Previous systematic reviews (Hao et al., 2017[6]; Karlsson and Saleh, 2017[14]; Yun et al., 2016[32]) assessed immune checkpoint inhibitors using interim results from early trials such as CheckMate 066 (Robert et al., 2015[21]) and CheckMate 067 (Wolchok et al., 2017[30]). However, updated long-term follow-up data, including outcomes up to 10 years, now provide more mature insights into safety and efficacy (Wolchok et al., 2024[31]). Earlier reviews did not include LAG-3 directed regimens in their comparative analyses, as their scope was limited to PD-1 and CTLA-4 inhibitors. Although PD-1 + LAG-3 therapy has been evaluated separately in advanced melanoma (Hasanzadeh et al., 2024[7]), this work does not place LAG-3 within a broader comparative context alongside other checkpoint classes. To address this gap, the present review provides a comprehensive synthesis of current immunotherapy strategies in advanced melanoma, including PD-1, CTLA-4 and LAG-3 regimens across monotherapy and combination approaches.

### Aim

This systematic review aims to evaluate and compare the efficacy (OS, PFS, ORR) and safety (grade ≥ 3 AEs) of immune checkpoint inhibitors, including PD-1 and CTLA-4 monotherapies, PD-1 + CTLA-4 and PD-1 + LAG-3 combinations in advanced unresectable melanoma.

### Research questions

1. Between PD-1 and CTLA-4 inhibitor monotherapies, which regimen demonstrates better efficacy and safety compared with traditional systemic therapies (e.g., chemotherapy, vaccines) in advanced unresectable melanoma?
2. Between the two combination regimens, PD-1 + CTLA-4 and PD-1 + LAG-3, which demonstrates superior efficacy and safety when compared against monotherapy?

## Methods

### Inclusion criteria

- **Population (P):** Adults with advanced unresectable or metastatic melanoma
- **Intervention (I):** Immune checkpoint inhibitors (ICI) targeting Programmed cell death protein-1 (PD-1), Cytotoxic T-lymphocyte associated protein-4 (CTLA-4), or Lymphocyte activation gene-3 (LAG-3) pathways, administered as monotherapy or in combination
- **Comparator (C):** Traditional therapies (e.g., chemotherapy, vaccines), monotherapy versus combination therapy
- **Outcomes (O):** Overall survival (OS), progression-free survival (PFS), objective response rate (ORR), and treatment-related adverse events (AEs)

Studies were included if they enrolled adults with advanced unresectable melanoma and evaluated immune checkpoint inhibitors (ICIs) as monotherapy or in combination, compared to traditional therapies or other ICI regimens. Eligible studies had to report at least one key clinical outcome: overall survival (OS), progression-free survival (PFS), objective response rate (ORR), or adverse events (AEs). Only English-language randomized controlled trials (RCTs) were considered. When multiple publications existed for the same trial, the most recent analysis was prioritized. Exclusion criteria included non-randomized studies, observational designs, case reports, reviews, adjuvant-only trials, or those lacking relevant survival or safety outcomes.

### Search strategy

Final systematic literature search was conducted on 18 March 2025 across PubMed and the Cochrane Central Register of Controlled Trials (CENTRAL) databases. Medical Subject Headings (MeSH) terms and related keywords were used, including “Melanoma,” “Programmed Cell Death 1 Receptor (PD-1),” “CTLA-4 Antigen,” “LAG-3 Protein,” as well as drug names such as “nivolumab”, “pembrolizumab”, “ipilimumab”, “tremelimumab”, and “relatlimab.” Boolean operators were applied to combine terms appropriately. The full search string was:

*(“Melanoma”[Mesh] OR melanoma[tiab]) AND ((“Programmed Cell Death 1 Receptor”[Mesh] OR PD-1[tiab] OR PD1[tiab] OR nivolumab[tiab] OR pembrolizumab[tiab]) OR (“CTLA-4 Antigen”[Mesh] OR CTLA-4[tiab] OR CTLA4[tiab] OR ipilimumab[tiab] OR tremelimumab[tiab]) OR (“Lymphocyte Activation Gene-3”[Mesh] OR LAG-3[tiab] OR LAG3[tiab] OR relatlimab[tiab]))*

Studies published from 2006 to 18 March 2025 (PubMed) and from database inception to 18 March 2025 (CENTRAL) were considered. The PubMed search, filtered for randomized controlled trials (RCTs), identified 213 records, while the Cochrane CENTRAL search identified 1,721 records after applying an English language filter.

### Study selection

**Figure 2:**
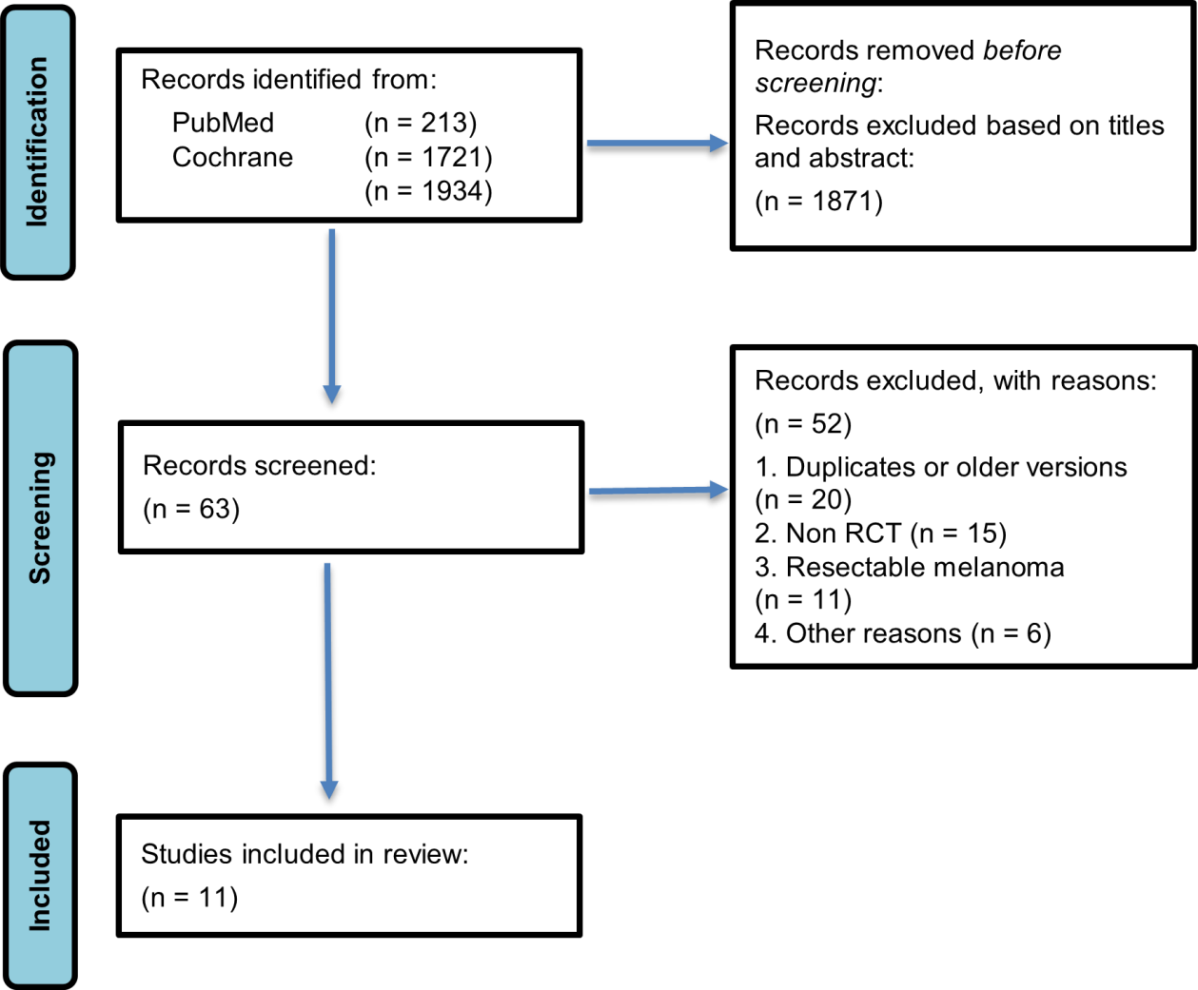
Flowchart of search and selection of studies (PRISMA flow diagram)

Following title and abstract screening, 63 studies were assessed in full text. During full-text screening, 52 studies were excluded for the following reasons: duplicate or older versions (n = 20), non-rct studies (n = 15), studies evaluating resectable melanoma (n = 11), and other reasons (n = 6). The selection process is summarized in the PRISMA flow diagram (Figure 2(Fig. 2)).

### Data extraction

Data extraction was conducted using a standardized form. Two summary tables were created: one described study and patient characteristics (study name, region, median age by arm, sample sizes, intervention/control treatments, ECOG performance status, and BRAF mutation status); the other summarized clinical outcomes. Outcomes were extracted separately for experimental and control arms, including median OS with 95 % CIs, HRs for OS and PFS, median PFS with 95 % CIs, ORR, and incidence of grade ≥ 3 AEs. Hazard ratios (HRs) for time-to-event outcomes quantify the relative rate at which events occur over time in the treatment group compared with the control group, whereas risk ratios (RRs) compare the overall probability of an event between groups. For trials with multiple publications, the most recent and complete datasets were prioritized to ensure mature data. Where key outcomes such as survival outcomes or AE profiles were incomplete, earlier interim reports were used to supplement missing information.

### Risk of bias assessment

Risk of bias was assessed using the Cochrane Risk of Bias 2.0 (RoB 2) tool, which evaluates five domains: Randomization process, deviations from intended interventions, missing outcome data, outcome measurement, and selection of reported results. Each domain was rated as “low risk,” “some concerns,” or “high risk” following Cochrane Handbook guidelines. An overall judgment was assigned based on domain-level ratings. Assessments were summarized narratively and tabulated using a color-coded system: green for low risk, yellow for some concerns, and red for high risk as detailed in Table 1(Tab. 1) (References in Table 1: Di Giacomo et al., 2024[2]; Hamid et al., 2017[5]; Hodi et al., 2010[10]; Hodi et al., 2016[9]; Larkin et al., 2018[15]; Ribas et al., 2013[19]; Robert et al., 2011[22]; Robert et al., 2020[20]; Tawbi et al., 2024[25]; VanderWalde et al., 2023[27]; Wolchok et al., 2024[31]).

### Data Synthesis

Data synthesis was conducted using Review Manager (RevMan) version 5.4.1. Hazard ratios (HRs) with 95 % confidence intervals (CIs) were pooled for time-to-event outcomes (OS and PFS), and risk ratios (RRs) for binary outcomes (ORR and grade ≥ 3 AEs). For all binary outcomes, raw event counts and total participants in each study arm were extracted and entered directly into RevMan, which calculated RRs using the Mantel-Haenszel random-effects model. A random-effects model was used to account for clinical and methodological heterogeneity. Statistical heterogeneity was assessed using the I^2^ statistic, with values > 50 % indicating substantial heterogeneity. Where meta-analysis was not feasible due to limited or inconsistent data, results were summarized narratively.

## Results

### Characteristics and quality assessment of included studies

A total of 11 randomized controlled trials were included in this systematic review and meta-analysis, as illustrated in the PRISMA flow diagram (Figure 2(Fig. 2)).

 The included studies enrolled adults with advanced unresectable or metastatic melanoma and evaluated immune checkpoint inhibitors as monotherapy or in combination. Three studies assessed CTLA-4 inhibitors (ipilimumab or tremelimumab) versus traditional therapies such as chemotherapy or vaccines. Three evaluated PD-1 inhibitors (nivolumab or pembrolizumab) against chemotherapy. Five investigated combination therapies: four assessed nivolumab plus ipilimumab (dual PD-1 and CTLA-4 blockade) versus ipilimumab alone, and one evaluated nivolumab plus relatlimab (dual PD-1 and LAG-3 inhibition) versus nivolumab. Sample sizes ranged from 53 to 714, with median ages typically between 56 and 64. Most studies included patients with ECOG performance status 0-1, and BRAF-mutated melanoma was present in 0-42 % of participants. Study characteristics are summarized in Table 2(Tab. 2) (References in Table 2: Di Giacomo et al., 2024[2]; Hamid et al., 2017[5]; Hodi et al., 2010[10]; Hodi et al., 2016[9]; Larkin et al., 2018[15]; Ribas et al., 2013[19]; Robert et al., 2011[22]; Robert et al., 2020[20]; Tawbi et al., 2024[25]; VanderWalde et al., 2023[27]; Wolchok et al., 2024[31]).

Risk of bias was assessed using the Cochrane Risk of Bias 2.0 (RoB 2) tool for RCTs, across five domains. Five studies (Robert et al., 2011[22]; Robert et al., 2020[20]; Hodi et al., 2016[9]; Wolchok et al., 2024[31]; Tawbi et al., 2024[25]) were rated low risk across all domains. Four (Ribas et al., 2013[19]; Larkin et al., 2018[15]; Hamid et al., 2017[5]; Di Giacomo et al., 2024[2]) were high risk, mainly due to deviations from intended interventions and lack of blinding or selective reporting. Two (Hodi et al., 2010[10]; VanderWalde et al., 2023[27]) had some concerns, primarily related to randomization or outcome assessment. Detailed assessments are presented in Table 1(Tab. 1).

### Efficacy

#### Overall survival (OS)

**Figure 3:**
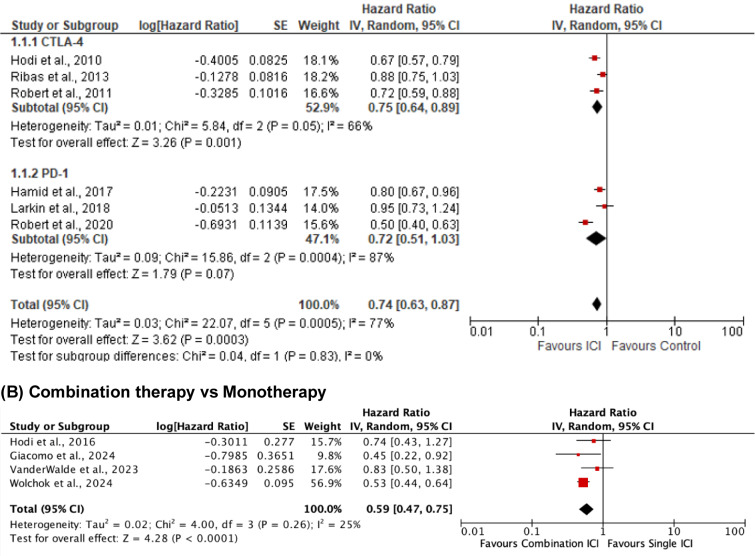
Overall survival (OS)

Ten randomized controlled trials involving 4,111 patients were included. Six studies (n = 3,196) comparing single ICIs to traditional therapies significantly improved OS in favor of ICIs (HR = 0.74; 95 % CI: 0.63-0.87; p = 0.0003), though heterogeneity was substantial (I^2^ = 77 %) (Figure 3(Fig. 3); References in Figure 3: Di Giacomo et al., 2024[2]; Hamid et al., 2017[5]; Hodi et al., 2010[10]; Hodi et al., 2016[9]; Larkin et al., 2018[15]; Ribas et al., 2013[19]; Robert et al., 2011[22]; Robert et al., 2020[20]; VanderWalde et al., 2023[27]; Wolchok et al., 2024[31]). Subgroup analysis showed benefit with CTLA-4 inhibitors (HR = 0.75; 95 % CI: 0.64-0.89; I^2^ = 66 %) and a favorable trend with PD-1 inhibitors (HR = 0.72; 95 % CI: 0.51-1.03; I^2^ = 87 %), though not statistically significant. The difference between subgroups was not significant (p = 0.83). Four additional trials (n = 915) comparing combination therapy to monotherapy demonstrated a clear survival advantage with dual ICIs (HR = 0.59; 95 % CI: 0.47-0.75; p < 0.0001), with low heterogeneity (I^2^ = 25 %) (Figure 3(Fig. 3)).

Three trials evaluated PD-1 inhibitors versus chemotherapy (Table 3(Tab. 3); References in Table 3: Di Giacomo et al., 2024[2]; Hamid et al., 2017[5]; Hodi et al., 2010[10]; Hodi et al., 2016[9]; Larkin et al., 2018[15]; Ribas et al., 2013[19]; Robert et al., 2011[22]; Robert et al., 2020[20]; Tawbi et al., 2024[25]; VanderWalde et al., 2023[27]; Wolchok et al., 2024[31]). Robert et al. (2020[20]) reported median OS of 37.3 months with nivolumab compared to 11.2 with dacarbazine (HR = 0.50; 95 % CI: 0.40-0.63). Hamid et al. (2017[5]) noted a modest benefit with pembrolizumab (14.05 vs. 11.0 months; HR = 0.80; 95 % CI: 0.67-0.96). In contrast, Larkin et al. (2018[15]) reported no significant difference (15.7 vs. 14.4 months; HR = 0.95; 95 % CI: 0.73-1.24).

Three trials assessed CTLA-4 inhibitors (Table 3(Tab. 3)). Hodi et al. (2010[10]) reported ipilimumab improved median OS to 10.1 months vs. 6.4 with gp100 (HR = 0.67; 95 % CI: 0.57-0.80). Robert et al. (2011[22]) showed improved OS with ipilimumab plus dacarbazine (11.2 vs. 9.1 months; HR = 0.72; 95 % CI: 0.59-0.87). Ribas et al. (2013[19]) reported no significant difference between tremelimumab and chemotherapy (12.6 vs. 10.7 months; HR = 0.88; 95 % CI: 0.75-1.04).

Four trials assessed PD-1 + CTLA-4 combinations versus ipilimumab alone (Table 3(Tab. 3)). Wolchok et al. (2024[31]) reported OS of 71.9 vs. 19.9 months (HR = 0.53; 95 % CI: 0.44-0.65), while Di Giacomo et al. (2024[2]) reported 29.2 vs. 8.2 months (HR = 0.45; 95 % CI: 0.22-0.91). Hodi et al. (2016[9]) and VanderWalde et al. (2023[27]) favored the combination, though results were not statistically significant (HR = 0.74 and 0.83, respectively). Tawbi et al. (2024[25]) evaluated PD-1 + LAG-3 (nivolumab + relatlimab) vs. nivolumab, reporting OS of 51.0 vs. 34.1 months (HR = 0.80; 95 % CI: 0.66-0.99).

#### Progression-free survival (PFS)

**Figure 4:**
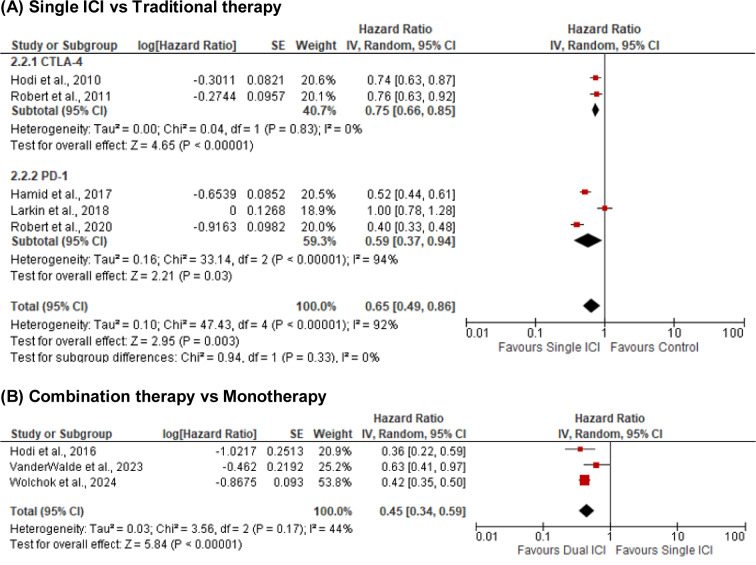
Progression free survival (PFS)

Eight randomized controlled trials involving 3,403 patients were included. Five studies (n = 2,541) comparing single ICIs to traditional therapies significantly improved PFS (HR = 0.65; 95 % CI: 0.49-0.86; p = 0.003), though heterogeneity was high (I^2^ = 92 %) (Figure 4(Fig. 4); References in Figure 4: Hamid et al., 2017[5]; Hodi et al., 2010[10]; Hodi et al., 2016[9]; Larkin et al., 2018[15]; Robert et al., 2011[22]; Robert et al., 2020[20]; VanderWalde et al., 2023[27]; Wolchok et al., 2024[31]). Subgroup analysis showed consistent benefit with CTLA-4 inhibitors (HR = 0.75; 95 % CI: 0.66-0.85; I^2^ = 0 %) and PD-1 inhibitors (HR = 0.59; 95 % CI: 0.37-0.94; I^2^ = 94 %), with no significant subgroup difference (p = 0.33). Three additional trials (n = 862) comparing dual ICIs to monotherapy reported a pooled HR of 0.45 (95 % CI: 0.34-0.59; p < 0.00001) with moderate heterogeneity (I^2^ = 44 %) (Figure 4(Fig. 4)).

Three studies evaluated PD-1 monotherapy (Table 3(Tab. 3)). Robert et al. (2020[20]) reported median PFS of 5.1 months with nivolumab vs. 2.2 with dacarbazine (HR = 0.40; 95 % CI: 0.33-0.54). Hamid et al. (2017[5]) reported similar benefit with pembrolizumab (HR = 0.52; 95 % CI: 0.44-0.62), though median PFS was not reported. Larkin et al. (2018[15]) reported no significant difference (3.1 vs. 3.7 months; HR = 1.00; 95 % CI: 0.78-1.44).

Two trials assessed CTLA-4 inhibitors (Table 3(Tab. 3)). Hodi et al. (2010[10]) reported slightly longer PFS with ipilimumab (2.78 vs. 2.76 months; HR = 0.74; 95 % CI: 0.63-0.87). Robert et al. (2011[22]) showed no difference in median PFS (2.8 months in both arms), though HR favored ipilimumab (0.76; 95 % CI: 0.63-0.93).

Five studies examined combination therapy (Table 3(Tab. 3)). Wolchok et al. (2024[31]) reported median PFS of 11.5 months with nivolumab + ipilimumab vs. 2.9 with ipilimumab alone (HR = 0.42; 95 % CI: 0.35-0.51). Di Giacomo et al. (2024[2]) showed a similar trend (8.7 vs. 3.3 months), though HR was not reported. Hodi et al. (2016[9]) and VanderWalde et al. (2023[27]) reported HRs of 0.36 and 0.63, respectively. Tawbi et al. (2024[25]). reported PFS of 10.2 vs. 4.6 months with PD-1 + LAG-3 vs. PD-1 alone (HR = 0.79; 95 % CI: 0.66-0.95).

#### Objective response rate (ORR)

**Figure 5:**
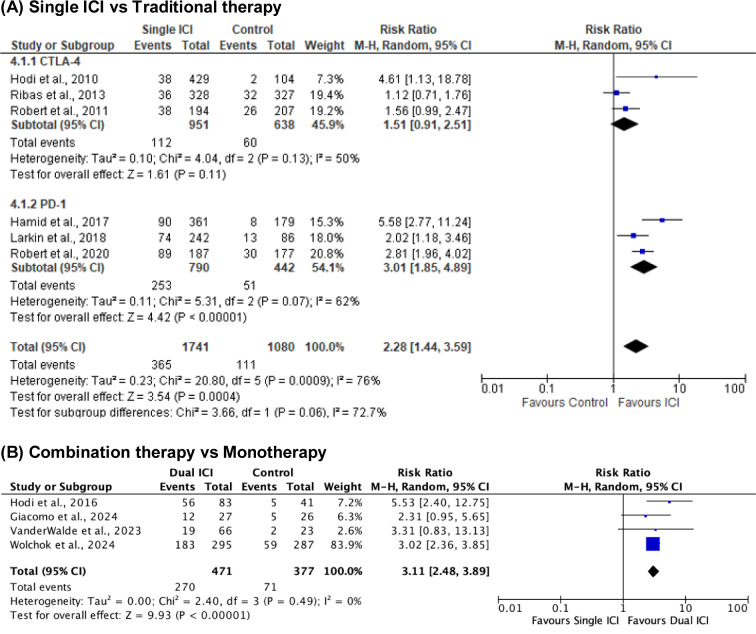
Objective response rate (ORR)

Ten randomized controlled trials involving 4,111 patients were included. Among six studies comparing single ICIs with traditional therapies, pooled analysis demonstrated significantly higher ORR with immunotherapy (RR = 2.28; 95 % CI: 1.44-3.59; p = 0.0004), though heterogeneity was substantial (I^2^ = 76 %) (Figure 5(Fig. 5); References in Figure 5: Di Giacomo et al., 2024[2]; Hamid et al., 2017[5]; Hodi et al., 2010[10]; Hodi et al., 2016[9]; Larkin et al., 2018[15]; Ribas et al., 2013[19]; Robert et al., 2011[22]; Robert et al., 2020[20]; VanderWalde et al., 2023[27]; Wolchok et al., 2024[31]). Subgroup analysis showed significant benefit with PD-1 inhibitors (RR = 3.01; 95 % CI: 1.85-4.89; I^2^ = 62 %), while CTLA-4 inhibitors did not reach statistical significance (RR = 1.51; 95 % CI: 0.91-2.51; I^2^ = 50 %). No significant subgroup difference was detected (p = 0.06).

Four trials comparing combination therapy to monotherapy reported significantly higher ORR with dual ICIs (RR = 3.11; 95 % CI: 2.48-3.89; p < 0.00001) with no heterogeneity (I^2^ = 0 %) (Figure 5(Fig. 5)).

Three trials evaluated PD-1 inhibitors (Table 3(Tab. 3)). Hamid et al. (2017[5]) reported an ORR of 25 % with pembrolizumab vs. 4 % with chemotherapy. Robert et al. (2011[22]) found 42 % with nivolumab vs. 14 %. Larkin et al. (2018[15]) reported 27 % vs. 10 %.

Three trials assessed CTLA-4 inhibitors (Table 3(Tab. 3)). Hodi et al. (2010[10]) reported 8.9 % with ipilimumab vs. 1.5 % with gp100. Ribas et al. (2013[19]) found similar response rates (11 % vs. 10 %). Robert et al. (2011[22]) reported 15.2 % vs. 10.3 % with ipilimumab plus dacarbazine vs. dacarbazine alone.

Five trials evaluated combination therapy (Table 3(Tab. 3)). Wolchok et al. (2024[31]) reported 58 % with nivolumab + ipilimumab vs. 19 %. Di Giacomo et al. (2024[2]) reported 44.4 % vs. 19.2 %. Hodi et al. (2016[9]) reported 59 % vs. 11 %, and VanderWalde et al. (2023[27]) 28 % vs. 9 %. Tawbi et al. (2024[25]) found 43.7 % vs. 33.7 % with PD-1 + LAG-3 vs. PD-1 alone.

### Safety

#### Grade ≥ 3 adverse events (AEs)

**Figure 6:**
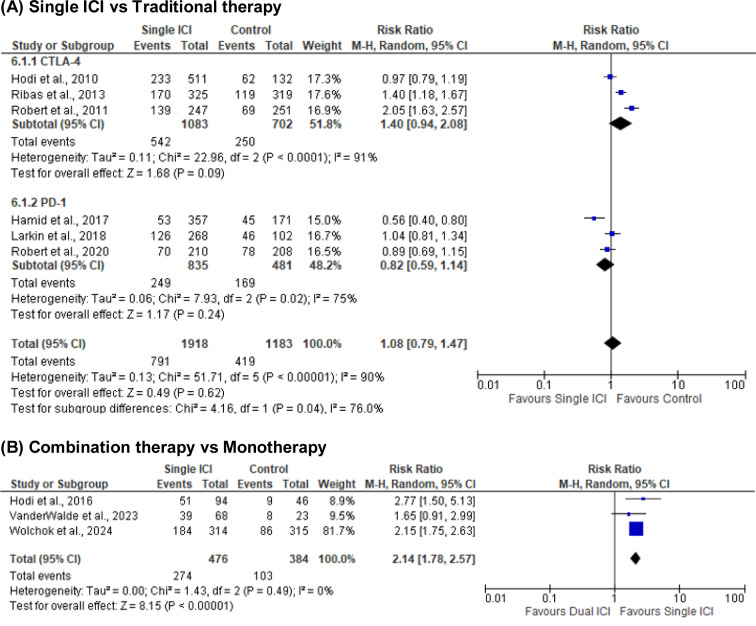
Adverse events (AEs)

Nine randomized controlled trials involving 4,058 patients were included. Six trials (n = 3,196) comparing single ICIs with traditional therapies reported no significant difference in grade ≥ 3 AEs (RR = 1.08; 95 % CI: 0.79-1.47; p = 0.62), with high heterogeneity (I^2^ = 90 %) (Figure 6(Fig. 6); References in Figure 6: Hamid et al., 2017[5]; Hodi et al., 2010[10]; Hodi et al., 2016[9]; Larkin et al., 2018[15]; Ribas et al., 2013[19]; Robert et al., 2011[22]; Robert et al., 2020[20]; VanderWalde et al., 2023[27]; Wolchok et al., 2024[31]). Subgroup analysis indicated higher, though non-significant, AE risk with CTLA-4 inhibitors (RR = 1.40; 95 % CI: 0.94-2.08; I^2^ = 91 %), and a non-significant trend toward lower risk with PD-1 inhibitors (RR = 0.82; 95 % CI: 0.59-1.14; I^2^ = 75 %). Subgroup difference was significant (p = 0.04), suggesting distinct toxicity profiles. Three trials comparing combination therapy vs. monotherapy reported significantly higher AE rates with dual ICIs (RR = 2.14; 95 % CI: 1.78-2.57; p < 0.00001), with no heterogeneity (I^2^ = 0 %) (Figure 6(Fig. 6)).

Three trials evaluated PD-1 inhibitors (Table 3(Tab. 3)). Hamid et al. (2017[5]) reported grade ≥ 3 adverse events in 15 % of pembrolizumab-treated patients vs. 26.3 % with chemotherapy. Robert et al. (2020[20]) found 34 % with nivolumab vs. 38 % for dacarbazine. Larkin et al. (2018[15]) reported a slightly higher rate with nivolumab (47 %) than chemotherapy (45 %).

Three trials assessed CTLA-4 inhibitors (Table 3(Tab. 3)). Hodi et al. (2010[10]) reported grade ≥ 3 AEs in 45.6 % with ipilimumab vs. 47 % with gp100 vaccine. Ribas et al. (2013[19]) observed 52 % with tremelimumab vs. 37 % with chemotherapy. Robert et al. (2011[22]) found 56.3 % with ipilimumab plus dacarbazine vs. 27.5 % with dacarbazine alone.

Five trials evaluated combination therapy (Table 3(Tab. 3)). Wolchok et al. (2024[31]) reported 59 % with nivolumab plus ipilimumab compared to 28 % with ipilimumab alone. Hodi et al. (2016[9]) observed 55 % vs. 19 %, VanderWalde et al. (2023[27]) reported 57 % vs. 35 %, and Di Giacomo et al. (2024[2]) found 52 % with the combination and 85 % with ipilimumab plus fotemustine. In contrast, Tawbi et al. (2024[25]) reported 46.2 % with nivolumab plus relatlimab versus 39.3 % with nivolumab alone, indicating a modest increase in toxicity, but lower than CTLA-4+PD-1 combination.

## Discussion

### Efficacy

Immune checkpoint inhibitors showed clear efficacy advantages over traditional treatments in advanced melanoma. Among monotherapy regimens, PD-1 inhibitors consistently demonstrated improved progression-free survival and objective response rates across trials. While the pooled overall survival result did not reach statistical significance, narrative synthesis of the included studies showed generally favorable outcomes with PD-1 treatment compared to control. This discrepancy may be explained by considerable heterogeneity among studies. In Hamid et al. (2017[5]) post-progression crossover to PD-1 therapy was allowed in the control arm, likely reducing the measurable survival difference between groups. Still, the consistency of direction across individual studies supports a meaningful clinical effect of PD-1 monotherapy. In contrast, CTLA-4 inhibitors showed more mixed results. While pooled analyses indicated significant improvement in PFS and OS, ORR did not reach statistical significance, and the results across trials were variable. Some studies showed only modest gains with CTLA-4 monotherapy, suggesting a less predictable therapeutic impact. These inconsistencies may reflect both clinical and biological factors, including trial population differences and the broader mechanism of immune activation associated with CTLA-4 inhibition. PD-1 and CTLA-4 inhibitors differ in their immune targets and functional effects, which may account for the differences observed in clinical performance. PD-1 blockade reactivates previously primed, antigen-experienced T cells within the tumor microenvironment, restoring localized cytotoxic immune activity against tumor cells. This targeted mechanism is more likely to produce sustained and tumor-specific responses. In contrast, CTLA-4 inhibition acts earlier in the immune response by promoting the expansion of naïve T cells in lymphoid tissues. While this broadens immune activation, it may result in less focused anti-tumor activity and contributes to the variability in treatment outcomes observed with CTLA-4 monotherapy (Buchbinder and Desai, 2016[1]).

Blocking two immune checkpoints simultaneously has emerged as a powerful strategy to improve treatment outcomes in advanced melanoma. Combination regimens consistently outperformed monotherapy across key efficacy endpoints, providing stronger and more durable clinical benefit. Dual inhibition of PD-1 and CTLA-4 produced particularly robust improvements, reflecting the synergy achieved by targeting different stages of the T-cell activation cycle. While PD-1 plus LAG-3 also demonstrated improved outcomes compared to PD-1 monotherapy, the survival advantage was statistically significant but more modest in scale. Nonetheless, both approaches underscore the therapeutic advantage of disrupting multiple immunosuppressive pathways rather than relying on a single mechanism.

The enhanced efficacy of PD-1 and CTLA-4 combination therapy reflects their complementary roles in immune regulation, targeting distinct stages of T-cell activation. This dual approach enables broader and more durable anti-tumor responses. In contrast, PD-1 and LAG-3 are often co-expressed on exhausted CD8+ T cells within the tumor microenvironment, where they suppress immune function through parallel pathways. LAG-3 primarily reduces cytotoxicity and cytokine production, while PD-1 limits T-cell proliferation. Blocking both pathways reactivates effector function by enhancing TCR signaling, increasing IFN-γ production, and restoring cytolytic activity. These mechanistic distinctions help explain why PD-1 + CTLA-4 blockade produces the most robust efficacy, while PD-1 + LAG-3 offers a more moderate, yet meaningful, clinical benefit (Rotte, 2019[23]; Qiu et al., 2024[17]).

### Safety

In terms of safety, the pooled analysis for grade ≥ 3 adverse events was not statistically significant in either the PD-1 or CTLA-4 subgroup. Both subgroups showed high heterogeneity, particularly CTLA-4. This likely reflects differences in trial design, especially background therapies. For instance, ipilimumab was combined with dacarbazine in Robert et al. (2011[22]) and with the gp100 vaccine in Hodi et al. (2010[10]), both of which are independently associated with toxicity and may have inflated adverse event rates in CTLA-4 arms. Narrative synthesis indicated that CTLA-4 monotherapy was more frequently associated with severe immune-related adverse events, whereas PD-1 inhibitors tended to be better tolerated across trials. As previously discussed, CTLA-4 blockade promotes broad immune activation by enhancing naïve T-cell priming in lymphoid tissues. This widespread stimulation increases the likelihood of off-target inflammation, particularly in barrier organs like the gastrointestinal tract, where immune-related colitis is common. In contrast, PD-1 inhibition acts locally within the tumor microenvironment, reactivating exhausted T cells without broadly stimulating the immune system. This tumor-specific reactivation contributes to the lower incidence of systemic immune-related adverse events observed with PD-1 (Wang et al., 2023[29]). Given these mechanistic differences, combining PD-1 and CTLA-4 blockade amplifies immune activation both during the early T-cell priming in lymphoid tissues (CTLA-4) and at the tumor site where PD-1 regulates exhausted T cells., which likely accounts for the significantly increased toxicity observed in pooled analyses. No heterogeneity was detected, suggesting this effect was consistent across studies. In contrast, PD-1 plus LAG-3 demonstrated a more favorable safety profile. As outlined previously, LAG-3 and PD-1 blockade acts more selectively on exhausted T cells in the tumor microenvironment, which may limit systemic immune activation. These findings support a clear toxicity gradient, with PD-1 in monotherapy being the most tolerable, followed by PD-1 + LAG-3 in the combination therapy (Wang et al., 2023[29]; Maruhashi et al., 2020[16]).

### Summary of findings

The findings of this systematic review and meta-analysis demonstrate that immunotherapy is more effective than traditional treatments in advanced melanoma. Single ICIs improved OS, PFS, and ORR, with PD-1 inhibitors consistently outperforming CTLA-4 inhibitors in both efficacy and safety. CTLA-4 inhibitors were associated with modest clinical benefits and a higher rate of severe adverse events, while PD-1 inhibitors showed greater improvements across all endpoints with a comparatively better safety profile. Combination therapy with PD-1 and CTLA-4 inhibitors provided the most substantial benefit in terms of survival and response, but this came with significantly increased toxicity. The PD-1 + LAG-3 combination, which represents a more recent approach targeting a novel immune checkpoint, demonstrated favorable efficacy while being better tolerated than PD-1 + CTLA-4. This suggests that PD-1 + LAG-3 may offer a more clinically viable option, especially for patients who may not tolerate the high toxicity associated with dual PD-1 and CTLA-4 blockade. These findings are consistent with previous studies reporting similar trends for PD-1 and CTLA-4 inhibitors (Hao et al., 2017[6]; Karlsson and Saleh, 2017[14]; Yun et al., 2016[32]).

### Strengths and limitations

The key strength of this systematic review with meta-analysis is the inclusion of only randomized controlled trials with generally low risk of bias. It captured the most widely used immune checkpoint inhibitors in melanoma, CTLA-4 and PD-1 as well as the newer PD-1 + LAG-3 combination, offering a broad and clinically relevant evaluation of current treatment strategies. The analysis included long-term follow-up where available, including studies with survival data up to 10 years, such as Wolchok et al. (2024[31]) enabling a more complete understanding of treatment durability. Efficacy and safety were comprehensively assessed through key outcomes including OS, PFS, ORR, and grade ≥ 3 AEs. Subgroup analyses enabled direct comparison across ICI classes.

While the findings are clinically meaningful and largely consistent with previous evidence, several limitations should be noted. Some outcomes, including OS for PD-1 monotherapy, did not reach statistical significance despite favorable trends. In Hamid et al. (2017[5]) two pembrolizumab doses (2 mg/kg and 10 mg/kg) were pooled, which may have introduced variability. Post-progression crossover occurred in multiple trials, including Hamid et al. (2017[5]) and Ribas et al. (2013[19]) potentially diluting treatment effects and underestimating survival benefit. Some monotherapy trials included ICIs with chemotherapy, such as ipilimumab with dacarbazine (Robert et al., 2011[22]) and nivolumab versus dacarbazine (Robert et al., 2020[20]), possibly confounding interpretation of ICI-only effects. Only 11 trials were included, limiting statistical power for subgroup analyses. Sensitivity analyses were not performed due to the small number of studies, and funnel plots were not generated, so publication bias could not be formally assessed. Heterogeneity was substantial in several analyses, particularly among single-agent comparisons, due to differences in trial design, treatment protocols, and patient populations. In the analysis of grade ≥ 3 AEs, the study by Di Giacomo et al. (2024[2]) was excluded as all other included studies were of high quality and showed consistent findings (I^2^ = 0 %), while Di Giacomo et al. (2024[2]) used an uncommon comparator (ipilimumab plus fotemustine) and reported a distinct toxicity profile, which would have introduced unnecessary heterogeneity. Lastly, the meta-analysis relied on published aggregate data rather than patient-level data, limiting the ability to conduct adjusted or stratified analyses.

### Future implications and research

The results of this systematic review and meta-analysis support the use of PD-1 inhibitors as the preferred monotherapy option in advanced melanoma, given their consistent efficacy and favorable safety profile compared to CTLA-4 inhibitors. Dual checkpoint blockade with PD-1 and CTLA-4 provides the most substantial survival benefit but is associated with high toxicity, which limits its use in many patients. The PD-1 + LAG-3 combination showed promising results with meaningful survival benefit and improved tolerability and may be a more suitable alternative for patients who are unable to tolerate the toxicity of PD-1 + CTLA-4 therapy.

Future research should focus on optimizing treatment sequencing, identifying reliable predictive biomarkers, and developing strategies to overcome resistance. Not all patients benefit from immune checkpoint inhibition, as these therapies only block certain immune pathways that may not be active in all tumors. A large proportion of patients continue to show no meaningful clinical response, even with combination therapy, which exposes them to unnecessary toxicity. This highlights the need to better understand why some patients do not respond and to expand treatment options beyond the currently available targets. Improving biomarker-based selection could help personalize therapy, reduce exposure to adverse effects in non-responders, and ultimately lead to better outcomes across a wider group of patients. Further clinical trials are also needed to evaluate LAG-3 based combinations across different melanoma subtypes, confirm their long-term benefit, and define their role within first-line treatment strategies.

## Conclusion

This systematic review with meta-analysis evaluated the comparative efficacy and safety of immune checkpoint inhibitors in advanced unresectable melanoma, including monotherapies and combination regimens across CTLA-4, PD-1, and LAG-3 pathways. The findings clearly demonstrate that immune checkpoint inhibitors offer superior efficacy compared to traditional therapies such as chemotherapy and vaccines, with improved overall survival, progression-free survival, and response rates. However, safety profiles varied. PD-1 inhibitors were associated with a lower incidence of grade ≥ 3 adverse events compared to traditional therapies, while CTLA-4 inhibitors, particularly ipilimumab, were linked to a higher rate of toxicity. Among monotherapies, PD-1 inhibitors provided the most favorable balance between efficacy and safety. Combination therapy with PD-1 and CTLA-4 yielded the most substantial improvement in efficacy but was associated with significantly increased toxicity, limiting its use in many patients. The PD-1 + LAG-3 combination showed promising results, with meaningful survival benefit and improved tolerability, suggesting it may serve as a more balanced alternative for patients unable to tolerate standard dual therapy. These findings directly address the clinical questions posed at the outset of this review and provide a clear comparative overview of current immunotherapy strategies in advanced melanoma.

## Declaration

### Preprint disclosure

A preprint version of this manuscript was previously published on the Qeios platform under the title “Efficacy and Safety of CTLA-4, PD-1 and LAG-3 Immune Checkpoint Inhibitors as Monotherapy and Combination Therapy in Advanced Melanoma: A Systematic Review and Meta-Analysis.” doi: https://doi.org/10.32388/4AAZYE.

The content has not been peer-reviewed elsewhere, and this submission represents the final, revised version intended for journal publication.

### Thesis declaration

This manuscript is based on the author's final-year dissertation completed as part of the Bachelor of Science (BSc) in Biomedical Science at the University of Buckingham, United Kingdom. The dissertation was submitted for academic evaluation only and has not been published elsewhere.

### Conflict of interest and funding

The author declares that they have no conflict of interest and no financial relationship with any organization that sponsored the research.

This work did not receive any specific grant from funding agencies in the public, commercial, or not-for-profit sectors.

### Ethical standards

This article does not contain any studies with human participants or animals performed by the author. All analyses were based on previously published data, and no new ethical approval was required. The study design, literature screening, data extraction, analysis, interpretation of results, and all scientific decisions were carried out entirely by the author. The author takes full responsibility for the content and conclusions of this work.

### Artificial Intelligence (AI) - assisted technology

Artificial intelligence tools were used solely to assist with language editing and improvement of clarity.

## Figures and Tables

**Table 1:**
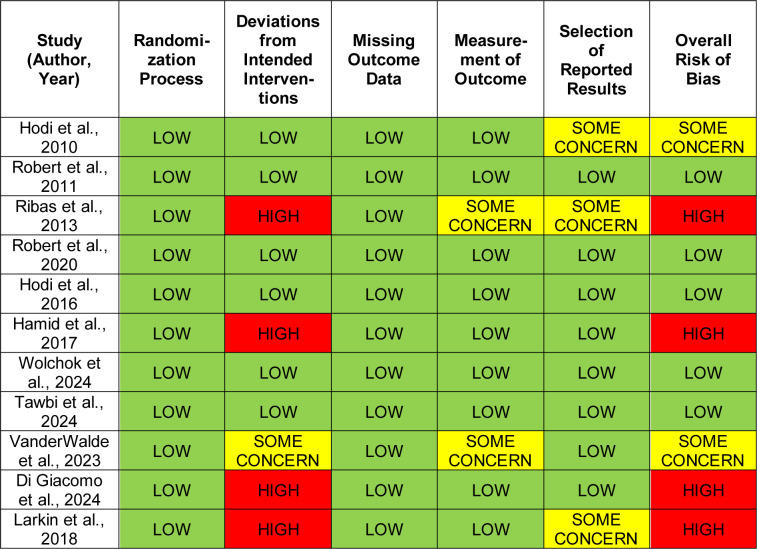
Risk of bias assessment

**Table 2:**
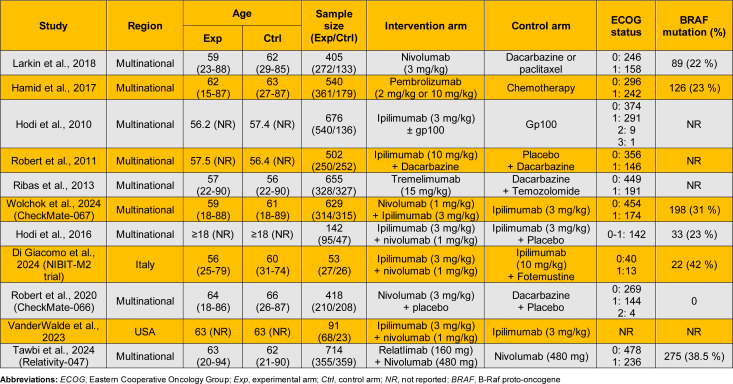
Characteristics of included studies

**Table 3:**
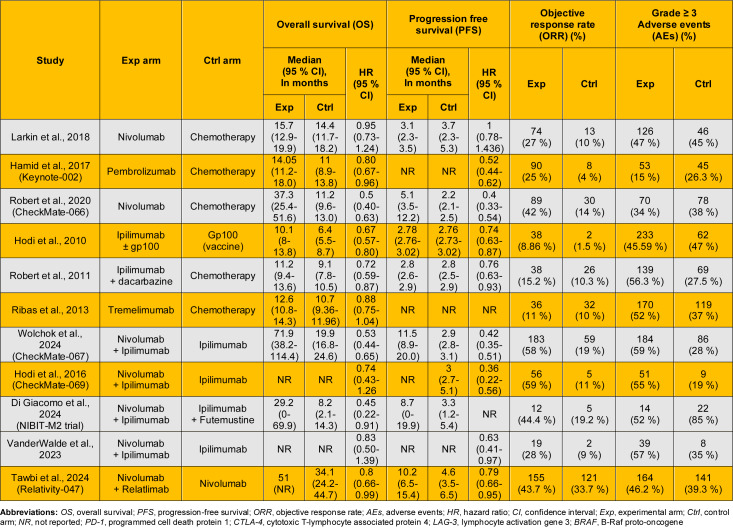
Safety and efficacy

**Figure 1:**
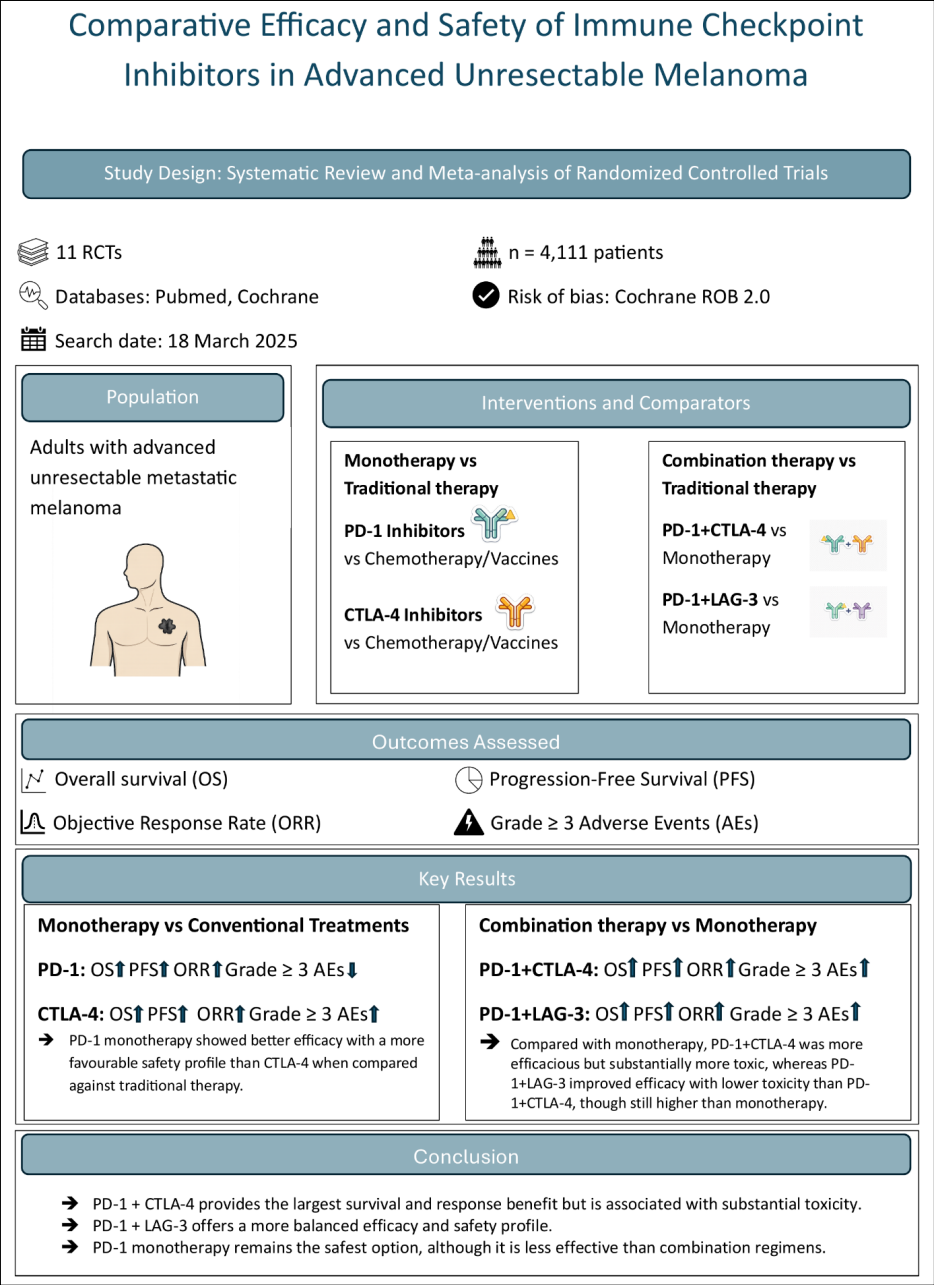
Graphical abstract
